# Distribution of allelic and genotypic frequencies of *NAT2* and *CYP2E1* variants in Moroccan population

**DOI:** 10.1186/s12863-014-0156-x

**Published:** 2014-12-29

**Authors:** Soukaina Guaoua, Ilham Ratbi, Fatima Zahra Laarabi, Siham Chafai Elalaoui, Imane Cherkaoui Jaouad, Amina Barkat, Abdelaziz Sefiani

**Affiliations:** Centre de génomique humaine, Faculté de médecine et de pharmacie, Université Mohammed V, Rabat, Morocco; Département de génétique médicale, Institut National d’Hygiène, Rabat, Morocco; Centre National de Référence en Néonatologie et en Nutrition, Rabat, Morocco

**Keywords:** Tuberculosis, *CYP2E1* gene, *NAT2* gene, Polymorphism, Acetylators, Adverse effects, Moroccans

## Abstract

**Background:**

Several pathogenesis and genetic factors influence predisposition to antituberculosis drug-induced hepatotoxicity (ATDH) especially for isoniazid (INH). However, the major susceptibility genes for ATDH are N-acetyltransferase 2 (*NAT2*) and cytochrome P450 2E1 (*CYP2E1*)*. NAT2* gene determines the individual’s acetylator status (fast, intermediate or slow) to metabolize drugs and xenobiotics, while *CYP2E1* c1/c1 genotype carriers had an increased risk of ATDH.

Polymorphisms of the *NAT2* and *CYP2E1* genes vary remarkably among the populations of different ethnic origins.

The aim of this study was to determine, for the first time, the frequency of slow acetylators in Moroccan population by genotyping of *NAT2* gene variants and determining the genotype c1/c1 for *CYP2E1* gene, in order to predict adverse effects of Tuberculosis treatment, particularly hepatotoxicity.

**Results:**

The frequencies of specific *NAT2* alleles were 53%, 25%, 2% and 4% for NAT2*5, NAT2*6, NAT2*7 and NAT2*14 respectively among 163 Moroccan studied group. Genotyping of *CYP2E1 *gene, by real-time polymerase chain reaction using TaqMan probes, revealed frequencies of 98.5% for c1/c1 and 1.5% for c1/c2 among 130 Moroccan studied group.

**Conclusion:**

The most prevalent genotypes of *NAT2* gene in Moroccans are those which encode slow acetylation phenotype (72.39%), leading to a high risk of ATDH. Most Moroccans are homozygous for c1 allele of *CYP2E1* gene which aggravates hepatotoxicity in slow acetylators.

This genetic background should be taken into account in determining the minimum dose of INH needed to treat Moroccan TB patients, in order to decrease adverse effects.

## Background

Pharmacogenetics refers to genetic differences in metabolic pathways which can affect individual responses to drugs, both in terms of therapeutic effect as well as adverse effects. Pharmacogenetics is generally regarded as the study or clinical testing of genetic variation that gives rise to differing responses to drugs. Its purpose is to optimize the therapeutic decisions based on the genome of the individual and the target molecule. Medicines are developed and used together with pharmacodiagnosis tools to achieve desired drug efficacy and safety.

Tuberculosis (TB) is an infectious disease caused by the *Mycobacterium tuberculosis.* TB remains to date one of the major public health problems in the world, with 8.6 million of incident cases, 12.0 million prevalent cases and 1.3 million deaths in 2012 [1]. In Morocco, 27,429 new cases of TB were reported in 2012, an incidence of 83 new cases per 100,000 inhabitants, according to the epidemiological services of the Moroccan Ministry of Health (Unpublished data).

The main drugs to treat TB are isoniazid (INH), rifampicin (RMP) and pyrazinamide (PZA), ethambutol (EMB) and/or streptomycin used in combination for 6 months or more [2]. Tuberculosis treatment cause adverse drug reactions (ADRs), including hepatitis, gastrointestinal intolerance, kidney failure, cutaneous and hematological reactions, which can lead to therapy discontinuation or more serious morbidity and mortality [3]. Among first-line anti-TB drugs, INH is the most effective but also the one that can easily cause hepatotoxicity.

The incidence of anti-tuberculosis drug-induced hepatotoxicity ranges from 1% to 36% [3-6]. Genetic factors have been reported as a risk for hepatotoxicity [7-10]. These factors were attributed to genetic variability in arylamine N-acetyltransferase2 (*NAT2)* gene, a cytosolic phase II conjugation enzyme primarily responsible for the deactivation of INH [7-11]. INH is metabolized to acetylisoniazid via hepatic NAT2 [12]. On the other hand, acetylisoniazid is hydrolyzed to acetylhydrazine, which is oxidized by cytochrome P450 2E1 (CYP2E1) to form some hepatotoxic intermediates [13,14]. Disposal of acetylhydrazine also depends on further acetylation by NAT2 to form a non-toxic metabolite, diacetylhydrazine [15,16].

*NAT2* gene on 8p22 is a key human enzyme in drug detoxification and elimination. Variants in *NAT2* gene affect the activity of anti-tuberculosis drugs and result in three different phenotypes: rapid (RA), intermediate (IA) and slow acetylators (SA). Most SNPs reported to date are found within the 873 bp intronless coding region of *NAT2* gene. Among the seven most common SNPs, four result in amino acid changes leading to a significant decrease in acetylation capacity and are associated to slow acetylator phenotype rs1801280 (c.341 T > C; NAT2*5), rs1799930 (c.590G > A; NAT2*6), rs1799931 (c.857 G > A; NAT2*7), and rs1801279 (c.191G > A; NAT2*14) [17,18]. The three others rs1041983 (c.282C > T; NAT2*13A), rs1799929 (c.481C > T; NAT2*11A), rs1208 (c.803A > G; NAT2*12A) are synonymous SNPs or do not alter the phenotype [17,18]. They have been identified as fast alleles [19,20].

NAT2*4 is considered as the reference allele in the case of absence of all the known SNPs, and is designated as a fast allele [19,20]. A heterozygous compound genotype (NAT2*4/*14 or NAT2*4/*5 or NAT2*4/*6 or NAT2*4/*7) is considered as intermediate acetylator. The pharmacogenetic interest of these data lies in adjusting the dose of isoniazid based on genotype and phenotype found in the patient in order to prevent hepatotoxicity [21].

In humans, the CYP2E1 enzyme is encoded by the *CYP2E1* gene on 10q24.3qter [22]. Various polymorphisms have been identified in the *CYP2E1* gene, of which the *CYP2E1 RsaI/PstI* polymorphism (rs2031920; -1053 C > T (Rsa 1 c1 > c2)) in its 50-flanking region may affect the activity or inductibility of the enzyme [23,24].

Polymorphisms of the *NAT2* and *CYP2E1* genes vary remarkably among the populations of different ethnic origins. Acetylators phenotypes in Moroccan population were previously reported to have a higher frequency of phenotypic slow acetylators [25].

The aim of this study was to determine the distribution of allelic and genotypic frequencies of *NAT2* and *CYP2E1* variants in Moroccan controls in order to estimate the prevalence of slow acetylators among Moroccans, who are facing the risk to develop hepatotoxicity after TB treatment.

## Results

In this study we identified twelve different genotypes for *NAT2* gene. The most of them encode for the slow acetylator phenotype. Genotypes of the four variants of *NAT2* gene and their corresponding phenotypic profiles are presented in Table 1. With regard to phenotype, the results show that 72.39% [95% CI 65.39-79.39] of the studied group were SA, 21.48% [95% CI 15.04-27.91] were IA and 6.13% [95% CI 2.38-9.88] were RA. For the four variants NAT2*5, NAT2*6, NAT2*7 and NAT2*14 of *NAT2* gene, the allelic frequencies in the studied group were 53% [95% CI 47-58], 25% [95% CI 20-29], 2% [95% CI 1-3] and 4% [95% CI 2-6], respectively (Table 2). *NAT2* alleles frequencies of Moroccans compared with other populations are shown in Table 2.

**Table 1:** **Observed frequency of** ***NAT2*** **genotypes encoding fast (RA), intermediate (IA) and slow (SA) acetylation phenotypes among Moroccan population studied**

**Genotype**	**Genotype frequency**	**Phenotype**
NAT2*4/*4	0.0613	RA
NAT2*4/*5	0.1166	IA
NAT2*4/*6	0.0859	IA
NAT2*4/*14	0.0123	IA
NAT2*5/*5	0.3313	SA
NAT2*5/*6	0.2209	SA
NAT2*5/*7	0.0184	SA
NAT2*5/*14	0.0368	SA
NAT2*6/*6	0.0797	SA
NAT2*6/*7	0.0184	SA
NAT2*6/*14	0.0123	SA
NAT2*14/*14	0.0061	SA

**Table 2:** **NAT2 alleles frequencies among Moroccan population and other ethnic groups**

**Ethnic groups**	**n**	NAT2*5	NAT2*6	NAT2*7	NAT2*14
**Caucasians [** 26 **]**	3531	0.46	0.285	0.029	0.00
**Germanians [** 27 **]**	844	0.425	0.278	0.013	0.001
**Americans [** 27 **]**	387	0.437	0.266	0.019	0.001
**Southern Korean [** 27 **]**	288	0.01	0.224	0.132	0.00
**Spanish [** 27 **]**	258	0.47	0.25	0.006	0.004
**Southern Brazil [** 28 **]**	254	0.289	0.104	0.021	0.014
**Egyptian [** 29 **]**	199	0.497	0.26	0.028	-
**Argentine [** 30 **]**	185	0.37	0.256	0.08	0.013
**Omanians [** 31 **]**	127	0.44	0.27	0.04	0.00
**Senegalians [** 27 **]**	101	0.322	0.188	0.00	0.084
**Tunisians [** 32 **]**	100	0.315	0.175	0.15	0.05
**South Africa [** 27 **]**	97	0.361	0.17	0.067	0.103
**Japanese [** 33 **]**	79	0.019	0.23	0.011	-
**Indians [** 33 **]**	61	0.33	0.38	0.03	-
**Moroccans (This study)**	163	0.53	0.25	0.02	0.04

**n**: sample size.

The allelic and genotypic distribution of the rs2031920 variant of *CYP2E1* in the studied group is reported in Table 3. The c2/c2 genotype was not found in the studied group. It seems that the majority of Moroccans are carriers of c1/c1 genotype with a frequency of 98.5% [95% CI 96-100]. *CYP2E1* genotypic frequencies of Moroccans compared with different populations of the world are shown in Table 4.

**Table 3:** **Allele and genotype frequencies of rs2031920 polymorphism in Moroccan controls**

	**Allele**		**Genotype**	
	**c1**	**c2**	**c1/ c1**	**c1/ c2**
Frequency	0.992	0.008	0.985	0.015

**Table 4:** **Genotypic frequencies of rs2031920 polymorphism of** ***CYP2E1*** **gene in different populations of the world**

	**Turkish [** 34 **]**	**Germanians [** 35 **]**	**Taiwanese [** 9 **]**	**Serbians [** 36 **]**	**French [** 37 **]**	**English [** 38 **]**	**Brazilians [** 28 **]**	**Chinese [** 26 **]**	**Indians [** 39 **]**	**Spanish [** 40 **]**	**Moroccans (This study)**
c1/c1	0.947	0.949	0.55	0.904	0.916	0.968	0.908	0.598	0.98	0.879	0.985
c1/c2	0.053	0.044	0.401	0.09	0.047	0.032	0.592	0.374	0.02	0.121	0.015
c2/c2	0.000	0.007	0.048	0.006	0.000	0.000	0.000	0.028	0.00	0.000	0.000
**n**	302	297	269	177	172	155	141	107	100	58	130

**n**: sample size.

## Discussion

This results show that slow acetylators are the most frequent in our Moroccan population, and the NAT2*5 allele is the most represented. Comparing the allele frequencies of the four *NAT2* variants reported in Table 2, the distribution pattern of NAT2*14 in Tunisian and NAT2*6, NAT2*7 in Caucasians populations did not vary significantly from our population (p > 0.05). On the other side, the allelic distribution in our population is different from other populations as Tunisians for the three alleles NAT2*5, NAT2*6 and NAT2*7 and from Caucasians for two alleles NAT2*5 and NAT2*14, this difference is statistically significant (p < 0.05) [26,32].

This difference with Tunisian neighbors could be explained by the origins of the Moroccan and Tunisian populations. Since about 8000 years ago native Berbers have been the major population group in all the North African regions, but through the centuries, Berbers have mixed differently with many other ethnic groups, Phoenicians, Carthaginians, Romans, Vandals, Byzantines, and Arabs whereas Ottoman rule reached Tunisia only [41,42]. Other explanation of the difference between our population and Tunisians could be a selection bias, and the limited size of their group controls.

Studies have shown variation in the distribution of *NAT2* alleles among different populations, where four major groups could be distinguished according to the frequency of NAT2*5 and NAT2*6 alleles and according to the presence of NAT2*7 and NAT2*14 alleles. NAT2*5 allele is the most common among Caucasian, Egyptian and Omanian populations as in our population [26,29,31], while Asians such as South Korean and Japanese populations, have less NAT2*5 and more NAT2*7 [26,27,33]. NAT2*6 variant is at the second position in our population similarly to Caucasians [26]. NAT2*14 allele, at the third position in our population, is rare in Caucasians and absent in Omanian and Southern Korea populations [26,27,31].

Junichi Azuma et al. 2012 [21], proposed in their recent study about *NAT2* genotypes and impact on doses of INH to increase the recommended dose of 5 mg/kg by the World Health Organization (WHO) to 7.5 mg/kg in rapid acetylators, maintain it in intermediate acetylators, and reduce it to 2.5 mg/kg in slow acetylators. As Moroccans are mainly slow acetylators, we propose according to our results to reduce the dose of INH in TB patients carrying slow acetylators genotypes, in order to prevent hepatotoxicity and to decrease the cost of managing adverse events.

For the polymorphism rs2031920 of the *CYP2E1* gene, our studied group consisted of 130 controls, originated from different regions of Morocco. From our results, it appears that there is a high frequency of c1/c1 genotype in Moroccan population (Table 3), which aggravates hepatotoxicity in slow acetylators patients under TB treatment. In Table 4, we compared our results with those of other reported populations. The frequencies of c1/c1, c1/c2 and c2/c2 genotypes in Moroccans were close to those found in Caucasians [35,37,38].

## Conclusion

In conclusion, this preliminary study shows that more than 70% of Moroccan subjects are carriers of NAT2*5, NAT2*6, NAT2*7 and NAT2*14 genotypes compatible with a slow acetylators status, and therefore, they are sensitive to lower doses of TB treatment. We should take into account this high prevalence of slow acetylators in order to decrease adverse effects, especially knowing that a vast majority of Moroccans are also homozygous for the c1 allele of *CYP2E1* gene, which aggravates hepatotoxicity.

## Methods

### Studied population

Blood samples were collected from umbilical cords of 163 unrelated newborns. They originated from different regions of Morocco and the Moroccan origin of their parents and grandparents was confirmed. Informed consent for DNA analysis was obtained from the parents. Ethics approval was obtained from the local committee of National institute of Hygiene in Rabat for this study.

### Genotyping protocol

Genomic DNA was extracted from three mL of blood using the salting-out method [43]. The quality and quantity of the DNA were controlled by A260/A280 using a Nanodrop spectrophotometer (2000/2000c Nanodrop; Fisher Scientific, Wilmington, DE, USA) and aliquots (50-100 μL) of packed blood cells were stored at 4°C until analyses. One hundred nanograms of extracted DNA was amplified in a final volume of 20 μL. Real time PCR mixture contained 10 μL of Master Mix (2X, TaqMan Genotyping Master Mix, Applied Biosystems), 0.5 μL of a specific probe (40 X, TaqMan, Applied Biosystems). The amplification protocol involves three steps, PCR which includes activation of Taq polymerase heating at 95°C for 10 minutes, followed by 40 cycles of amplification of 75 seconds each cycle (DNA denaturation for 15 seconds at 92°C, Hybridization for 1 minute at 60°C) and ends with Post-PCR Read at 60°C for 1 minute.

We genotyped 163 DNA samples for the four SNPs with strongest impact on the acetylation profile: rs1801280 (c.341 T > C; NAT2*5), rs1799930 (c.590G > A; NAT2*6), rs1799931 (c.857 G > A; NAT2*7), and rs1801279 (c.191G > A; NAT2*14) polymorphisms of *NAT2* gene and 130 DNA samples for the rs2031920 polymorphism of *CYP2E1* gene.

Genotyping of SNPs of both genes was performed with an allele-specific probe of SNP using allele-specific real-time polymerase chain reaction (StepOne Real-Time PCR System; Applied Biosystems7500, Foster City, CA, USA) using TaqMan (Applied Biosystems, Warrington, UK) probes. This method combines PCR and mutation detection in a single step. A hybridization probe is cleaved by the 5’ nuclease activity of *Taq* DNA polymerase only if the specific sequence is successfully amplified. Two TaqMan probes are used, one for each allele. TaqMan probes consist of a 18–22 bp oligonucleotide probe which is labeled with a reporter fluorophore at the 5’ end and a quencher fluorophore at the 3’ end. Allelic discrimination was obtained by the post-PCR read of fluorescence intensity.

For the *CYP2E1* polymorphisme the primers sequences were from assays-by-designs ^SM^ of the manufacturer’s (Applied Biosystems). Alleles of rs2031920 were assessed using primers rs2031920_F: TGACTTTTATTTTCTTCATTTCTCATCATATTTTCTATTATACAT and rs2031920_R: GTTTTTCATTCTGTCTTCTAACTGGCAATAT and the Taqman probes rs2031920_V: VIC AGGTTGCAATTTT*G*TACTTT and rs2031920_F: FAMGTTGCAATTTT*A*TACTTT (SNP position highlighted).

For the four polymorphisms of *NAT2* gene, the primers sequences were from Drug metabolism genotyping assay of the manufacturer’s (Applied Biosystems) which reference is (p / n 4362038).

Genotype frequencies in our population were calculated in accordance with the Hardy-Weinberg equilibrium. Intervals confidence 95% were calculated for phenotypic genotypic and allelic frequencies.

### Statistical analysis

Statistical analysis was performed using the SPSS (Statistical Package for the Social Sciences) version 17.0 for windows. The chi-square test was used to determine whether there is a significant difference between the expected frequencies and the observed frequencies relative to *NAT2* gene distribution in the studied group versus other populations. Statistical significance was assumed at the p < 0.05.

## Abbreviations

ATDH: Antituberculosis drug-induced hepatotoxicity
INH: Isoniazid
*NAT2*: N-acetyltransferase 2
*CYP2E1*: Cytochrome P450 2E1
TB: Tuberculosis
INH: Isoniazid
RMP: Rifampicin
PZA: Pyrazinamide
EMB: Ethambutol
ADRs: Adverse drug reactions
RA: Rapid acetylator
IA: Intermediate acetylator
SA: Slow acetylator
WHO: World Health Organization
SPSS: Statistical package for the social sciences
n: Sample size
